# Headache Attributed to Refractive Error: Improvement after Topography-Guided Photorefractive Keratectomy with Corneal Cross-Linking in Patients with Keratoconus

**DOI:** 10.3390/jcm13030690

**Published:** 2024-01-25

**Authors:** Nigel Terk-Howe Khoo, Barbara Burgos-Blasco, Angelique Antoniou, Bronwyn Jenkins, Clare L. Fraser, Gregory Moloney

**Affiliations:** 1 Save Sight Institute, Faculty of Health and Medicine, The University of Sydney, Sydney, NSW 2050, Australia; 2 Ophthalmology Department, University of British Columbia, Vancouver, BC V5Z 1J9, Canada; 3 Narellan Eye Specialists, Narellan, NSW 2567, Australia; 4 Royal North Shore Hospital, Sydney, NSW 2065, Australia; 5 Discipline of Ophthalmology, Sydney Medical School, The University of Sydney, Sydney, NSW 2006, Australia; 6 Sydney Eye Hospital, Sydney, NSW 2000, Australia

**Keywords:** headache, refractive error, topography guided photorefractive keratectomy, PRK, corneal cross-linking, keratoconus

## Abstract

Purpose: To investigate if topography-guided photorefractive keratectomy (TGPRK) alleviates headache, particularly headache attributed to refractive errors (HARE) in keratoconus. Methods: Patients diagnosed with keratoconus undergoing TGPRK for refractive correction were included. Best spectacle corrected visual acuity (BSCVA) using the logMAR scale and refractive error were measured. Patients answered a questionnaire exploring headaches, characteristics, treatment, and the Headache Impact Test (HIT-6) before and 6 months after the surgery. Results: 40 patients were included. Preoperatively, 24 patients (60%) met criteria for headaches: five for migraine, 14 for HARE, and five for tension-type headache (TTH). Patients with headaches preoperatively were more likely to require bilateral TGPRK, and the mean sphere and cylindrical power were higher. Postoperatively, 15 out of the 24 patients of the headache group experienced complete resolution of headaches, and only nine patients met diagnostic criteria for headaches: two for migraine, six for HARE, and one for TTH. The number of headaches reduced from 4.4 ± 2.4 to 0.5 ± 0.7 days/week (*p* < 0.001). Headache duration decreased from 108.5 ± 100.7 min to 34.4 ± 63.5 min (*p* = 0.002). Postoperatively, the consumption of analgesia decreased. The HIT-6 revealed an improvement in the quality-of-life post-procedure (*p* < 0.001). Conclusions: Surgical correction of irregular astigmatism in patients with keratoconus can alleviate or resolve headaches in a large proportion of patients, resulting in an improvement in their quality of life. Physicians should consider keratoconus in patients fitting criteria for HARE not alleviated by spectacle correction and suboptimal vision in glasses.

## 1. Introduction

Headache disorders are highly debilitating and affect millions worldwide, with a considerable impact on a patient’s quality of life. Every day, one million people across Europe suffer from migraine attacks, and this has a tremendous economic impact that has been estimated to be several billion dollars per year. Primary headache disorders cause approximately 20% of all absenteeism due to sickness. Despite this, headache has never been the subject of any national public health project in any country, perhaps because of its episodic nature and lack of mortality [1,2,3].

The International Headache Society (IHS) recognizes refractive error as a cause of headache in Section 11.3.2 of the third edition of The International Classification of Headache Disorders, under ‘Headache Attributed to Refractive Error (HARE) [4]. They are defined as headaches that have developed or worsened in temporal relation to the onset or worsening of the refractive error, improve after correction of the refractive error, are aggravated by prolonged visual tasks at the distance at which vision is impaired, or improve when the visual task is discontinued.

Nonetheless, the role of uncorrected refractive errors such as myopia, hyperopia, and astigmatism in headaches remains controversial. In the 20th century, the link between refractive error and headache came from anecdotal clinical evidence [5]. In 1943, Eckardt et al. found an association between refractive error and headache [6]. Subjects who wore lenses simulating 5D of hypermetropia complained of ocular discomfort after a short period of time, and this was exacerbated by tasks requiring near vision. Indeed, some authors have described a possible significant association between these two conditions, while other authors have justified this link by their high prevalence in the general population [7,8,9,10].

The mechanism or pathophysiology of headache in patients with refractive error remains unclear, although there are several theories. The hypothesis of ciliary muscle spasm or fatigue has been widely accepted for the last century [11]. Other proposed theories include that exaggerated muscular effort of the occipitofrontalis, extrinsic eye, or corrugator supercilii muscles required to compensate for myopia (“brow furrowing”) may provoke headaches [12,13].

Keratoconus is a corneal ectatic disorder in which the cornea becomes progressively thinner, eventually assuming a cone-like shape resulting in progressive myopia and astigmatism. The pathogenesis of keratoconus is felt to involve the interplay of genetic susceptibility and environmental triggers, chiefly mechanical strain in the form of eye rubbing, or pressure during sleep [14,15]. In the presence of ongoing mechanical stimulus, untreated keratoconus will result in progressive worsening of visual quality to debilitating levels.

Initial non-surgical interventions in the correction of refractive error in patients with keratoconus include conventional spectacles and contact lenses [16]. However, patients with keratoconus have an irregular corneal shape, so spectacles or soft contact lenses may not be adequate options to correct refractive error, and patients may still experience poor quality of vision due to high-order aberrations [17,18]. Glasses cannot be designed to correct for irregularity in the wavefront, and soft contact lenses will assume the irregular shape of the cornea they are draped over. Rigid gas permeable lenses or scleral lenses are the only non-surgical options that can overcome irregular astigmatism but are variably tolerated by patients.

Surgical techniques used to correct refractive error in keratoconus include topography-guided photorefractive keratectomy (TGPRK), intracorneal ring segments (plastic or allogenic tissue), phakic lenses (Implantable Collamer Lens -ICL or ArtiLens), pseudophakic lenses, or corneal transplantation. TGPRK is a form of surface-based excimer laser ablation in which a treatment profile is generated based on the patient’s corneal shape as well as their refraction, with the goal being to transform an irregular cornea into a regular shape, correctable with glasses. This is usually combined with cross-linking (CXL), which stabilizes the collagen fibers of the cornea, preventing the progression of the disease [19,20,21,22].

Current evidence investigating the link between keratoconus and headache in the literature is limited. Previous studies have attempted to explore this, at times with variable classification systems or analysis of heterogenous headache subtypes, highlighting the difficulties in analyzing the association [4,10]. Furthermore, there have not been any previously performed studies investigating headaches following the surgical correction of refractive error in keratoconus by means of TGPRK and CXL.

Given that patients with keratoconus commonly present with refractive error that cannot be corrected with glasses or contact lenses due to the presence of high-order aberrations, it is possible that patients with keratoconus have a higher risk of HARE and are not accessing appropriate methods of refractive correction—either rigid contact lenses or surgical options. Therefore, we aimed to investigate whether surgical correction of refractive error in keratoconus with TGPRK alleviates headache frequency and medication use from an appropriately classified patient population group.

## 2. Methods

An observational study, including patients diagnosed with keratoconus undergoing TGPRK for refractive correction, was performed. This project was granted ethics approval by the Human Research Ethics Committee of the University of Sydney and adhered to the tenets of the Declaration of Helsinki. All participants provided informed consent.

Patients with a diagnosis of keratoconus in at least one eye undergoing TGPRK with CXL between January 2017 and December 2019 were included. TGPRK was offered to patients with poor vision not correctable with glasses or soft contact lenses and who were intolerant of rigid lenses. All procedures were performed on the Schwind Amaris 1050 Hz system, with topographic data are collected from the Sirius topographer and exported to the custom ablation manager software for analysis and planning A residual stromal bed of greater than 300 µm was planned for each case. Exclusion criteria were: known co-morbidities for headache such as intracranial hypertension or other ophthalmic pathology that could cause eye strain or headache, including heterophoria or glaucoma. All participants underwent a review of past medical history and an ophthalmic examination to confirm the inclusion and exclusion criteria.

Before surgery, a general and ocular history was taken, and the following variables were collected: age, sex, presence of systemic diseases, previous ocular surgeries, and previous ocular diseases. Then, best spectacle corrected visual acuity (BSCVA) using the logMAR scale and refractive error were measured. An anterior segment slit-lamp examination was also performed, and abnormal findings were noted. Pentacam HR (Oculus Optikgeräte GmbH, Wetzlar, Germany) was used for corneal topography in order to confirm the diagnosis of keratoconus.

In addition, patients were given two questionnaires. The first questionnaire explored headache, characteristics, and its treatment in the participants and was developed by a neurologist specialized in headaches (Appendix A). The second questionnaire was the Headache Impact Test (HIT-6), which has proven effective in measuring the impact of headache on patients’ quality of life. The four headache impact severity categories are little or no impact (49 or less), some impact (50–55), substantial impact (56–59), and severe impact (60–78) [23,24].

PRK was performed using the Schwind excimer laser (500 Hz), and all ablations were transepithelial with no manual removal of epithelium. CXL was performed immediately after. Hypotonic riboflavin was used, and UV-A was delivered with 3.2 J total energy delivered at an 18 mw/cm^2^ irradiance and a pulsed duty cycle. Mitomycin C 0.02% was applied for 40 s, followed by irrigation with a balanced salt solution. After the procedure, a bandage contact lens was applied upon completion of the procedure, and the patient was prescribed ofloxacin eyedrops 3 mg/mL four times daily for one week and dexamethasone 0.1% eyedrops two times daily until contact lens removal, then four times daily for two weeks, and tapering over six weeks.

Postoperative BSCVA, refractive error, and questionnaire scores were also collected at six months postoperatively.

Myopia was defined as the spherical equivalent refraction of at least −0.50 D, astigmatism as the cylinder of at least 1.0 D, and anisometropia as the spherical difference of at least 2.0 D between two eyes of the same patient. Patients who required bilateral TGPRK and CXL were classified according to the more myopic eye. Compound astigmatism was defined as the condition in which the two principal meridians of an eye are either myopic or hyperopic. Mixed astigmatism was defined as the condition in which one meridian is hyperopic, while the one at a right angle to it is myopic.

HARE was defined as a headache that has developed or worsened in temporal relation to the onset or worsening of the refractive error, improves after correction of the refractive error, is aggravated by prolonged visual tasks at the distance at which vision is impaired, or improves when the visual task is discontinued. Migraine and TTH fall under the subset of primary headache disorders. In Section 1, the IHS divides migraine into two main types—migraine with aura and migraine without aura. Migraine without aura is defined as a recurrent headache disorder manifesting in attacks lasting 4–72 h. Typical characteristics of migraine are unilateral location, pulsating quality, moderate or severe intensity, aggravation by routine physical activity, and association with nausea and/or photophobia and phonophobia. The IHS describes TTH in Section 2 of primary headache disorders. TTH is divided into four main categories—infrequent episodic TTH, which is the most common in the general population, frequent episodic TTH, chronic TTH, and probably TTH. If TTH is attributed to another disorder, the diagnosis will need to be coded to the other disorder.

Statistical analysis was performed using SPSS version 26.0 (SPSS, Chicago, IL, USA). Quantitative variables are represented by their mean and range, while qualitative variables are shown as numbers and percentages. Differences between the groups (headache and non-headache) were investigated using Student’s *t*-test, paired-*t* test, and McNemar’s test, where appropriate. *p* < 0.05 was considered statistically significant.

## 3. Results

Of the initial 60 patients that were screened, 40 patients met the inclusion criteria and agreed to take part in the study. The study sample comprised 28 males and 12 females. Overall, the average preoperative BSCVA improved from 0.4 ± 0.3 to 0.1 ± 0.2 logMAR.

### 3.1. Preoperative Characteristics of Headache and Non-Headache Groups

Preoperatively, 24 patients (60%) met criteria for headaches (headache group), five of which met criteria for migraine, 14 for HARE, and 5 for tension-type headache (TTH). The mean number of months of headaches prior to surgery was 48.3 ± 80.9 months (range 2–360 months). 16 patients (40%) were headache-free (non-headache group) preoperatively. The mean age in the pre-existing headache group was 34.9 ± 11.2 (range of 18–55) and 35.9 ± 11.1 (range of 21–55) in the non-headache group (*p* = 0.780).

17 patients underwent unilateral TGPRK and CXL, and 23 patients had bilateral sequential TGPRK and CXL. No patient underwent bilateral same-day TGPRK with a minimum of one month between treatments. Patients with headaches preoperatively were more likely to require bilateral TGPRK and CXL (20 patients, 87% of headache group). Conversely, only three patients in the non-headache group underwent bilateral TGPRK and CXL. 14 patients out of 16 in the non-headache group underwent unilateral TGPRK and CXL (*p* < 0.001). In addition, patients who had unilateral TGPRK and CXL experienced a mean of 2.0 ± 2.6 days of headache per week, compared with 4.4 ± 2.8 headache days per week in the group of patients who required bilateral TGPRK and CXL (*p* = 0.015).

The prevalence of severe astigmatism was higher in the headache group (29.2%) compared to the non-headache group (18.8%; *p* = 0.453). The prevalence of moderate myopia was also higher in the overall headache group: 45.8%, compared to 18.8% in the non-headache group (*p* = 0.039). However, the prevalence of anisometropia was 45.8% and 50.0% in the overall headache and non-headache groups, respectively (*p* = 0.794). Detailed data about the classification of refractive errors in the overall headache and non-headache groups are presented in Table 1.

### 3.2. Postoperative Characteristics of Headache and Non-Headache Groups

Postoperatively, only nine patients met IHS diagnostic criteria for a headache disorder, compared to 24 preoperatively. Of these nine patients, two met criteria for migraine, six for HARE, and one for TTH.

### 3.3. Differences in Headache Diagnosis, Characteristics, and Treatment after Surgery

#### 3.3.1. Headache Diagnosis According to IHS Criteria

Postoperatively, 15 out of the 24 patients of the headache group experienced complete resolution of headaches (0 headache days/week). None of the patients in the non-headache group developed new headaches postoperatively.

#### 3.3.2. Headache Days and Duration

The changes in number and duration of headaches are presented in Table 2. Preoperatively, in the overall headache group, the mean number of headache days per week was 4.4 ± 2.4 days (range 1–7). Postoperatively, this reduced to 0.5 ± 0.7 days/week (range 0–3; *p* < 0.001). Moreover, the mean duration of the headaches decreased from 108.5 ± 100.7 min (range 10–360) to 34.4 ± 63.5 min (range 0–240; *p* = 0.002).

Regarding the types of headaches, the overall trend was a decrease in the mean number of headache days per week, along with a decrease in the mean headache duration. The largest reduction in the number of headache days/week was seen in the HARE group, while patients in the migraine subgroup experienced the largest reduction in the duration of headaches postoperatively (Table 2).

#### 3.3.3. Analgesia Use

**Figure 1:**
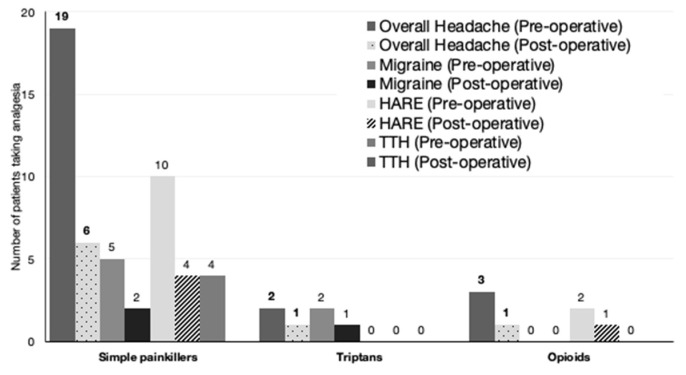
Decrease in the number of patients taking analgesia in the overall headache and headache subgroups. HARE: headache attributed to refractive error, TTH: tension-type headache.

In the preoperative overall headache group, 19 patients consumed simple analgesia at a mean of 2.4 ± 2.3 days a week (range of 1–7 days). A further five patients consumed additional analgesia: two patients took migraine relief medication such as triptans, and another three patients consumed opioids (Figure 1).

**Figure 2:**
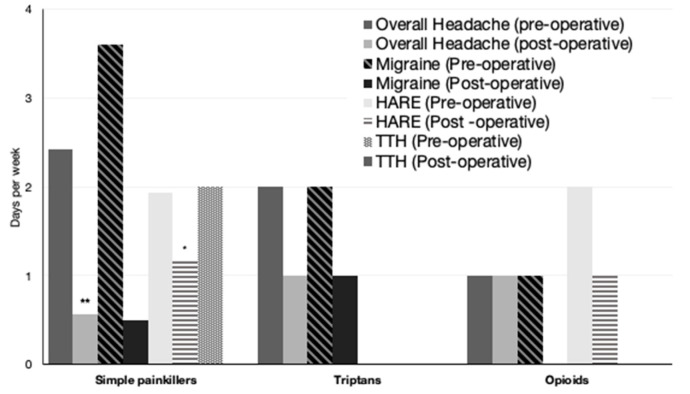
Decrease in the overall consumption of painkillers postoperatively in the overall headache group and headache subgroups. HARE: headache attributed to refractive error, TTH: tension-type headache. * indicates *p* < 0.05 compared to the preoperative value, ** indicates *p* < 0.01 compared to the preoperative value.

Postoperatively, six out of the nine patients with headaches consumed simple painkillers, averaging 0.6 ± 1.2 days a week, significantly lower than 2.4 ± 2.3 days a week preoperatively (Figure 2) (*p* < 0.001). This analysis includes patients who no longer had headaches postoperatively.

#### 3.3.4. Headache Impact and HIT-6 Score

The headache impact questionnaire revealed an overall improvement in the quality of life post-procedure. When asked about changes compared to headaches prior to surgery, patients in the headache group reported a subjective improvement of 82.1 ± 19.6% (range 10–99) in terms of frequency and severity of headaches combined, 82.4 ± 19.5% (range 10–99) in frequency alone, and 83 ± 19.28% (range 10–99) in severity of headaches alone.

The preoperative mean HIT-6 score was 62.4 ± 10.1 (range 40–78), which improved significantly postoperatively to 46.6 ± 13.9 (range 36–78; *p* < 0.001, Table 3). Table 4 summarizes the improvement of HIT-6 scores across all headache subgroups.

## 4. Discussion

Ophthalmologists can play an important role in the management of headaches, as Ophthalmology is the third most consulted specialty for headaches of recent onset. Among different types of headaches, HARE may be corrected or improved if the refractive error is treated. Keratoconus patients are of particular interest because they may be at increased risk of HARE due to the presence of high-order aberrations that cannot be corrected with glasses or contact lenses. The present findings show that patients with keratoconus who underwent correction of refractive error by means of TGPRK and CXL found a significant alleviation of headaches, as evidenced by the reduction in headache impact scores, analgesia consumption, and the number of headache days per week.

The frequency of HARE is still debated. Several groups have found that migraine patients have higher degrees of astigmatic refractive error, spherical equivalent, and anisometropia compared to non-headache subjects [8,25]. As for HARE in children, the prevalence of refractive errors has been found to be higher in headache patients, as well as the rate of astigmatism, anisometropia, and previous miscorrection of refractive error [9]. In another study involving 487 children, 70% of them reported the occurrence of headaches in the last year. In this case, the association between headache complaints and the sphere component was statistically significant but small [26]. Harle and Evans evaluated the correlation between headaches and refractive variables, but the correlations between the severity of the worst headache, duration of the worst headache, days since the last migraine headache, and mean sphere, mean astigmatic power, anisometropia, and uncorrected error were all low and not significant [27]. In the most recent study on adults, Lajmi et al. identified the following independent risk factors for HARE: prolonged exposure to the screen, the complex nature of ametropia, moderate hyperopia, and moderate astigmatism [7].

Investigations addressing the prevalence of headache and primary headache disorders in keratoconus patients have not been specifically addressed. In our series, 24 of 40 patients (60%) experienced headaches preoperatively, suggesting that headaches are a common occurrence among patients with keratoconus for whom standard methods of refractive correction have proved ineffective.

Within our cohort, those requiring bilateral TGPRK and CXL were more likely to suffer from headache preoperatively. Conversely, those with unilateral disease were less affected by headaches, with the prevalence of anisometropia highest in the non-headache group preoperatively. Ophthalmologists are used to anisometropia presenting as a source of visual confusion and discomfort. It was to our surprise, therefore, that this was not associated with increased headaches in this study. We hypothesize that bilateral disease with no eye providing a useful focused image may lead to increased “search for focus”. This will result in ciliary muscle overactivity or squinting/furrowing of the brow with resultant increased tension-type headache. It seems that in unilateral keratoconus, patients may be able to suppress the defocused image and use the better eye, with relaxation of the aforementioned muscle groups.

After surgery for the correction of refractive error, 15 of the 24 patients in the headache group experienced complete resolution of headaches. There was a decrease in the mean number of headache days per week, the mean duration of the headaches, and analgesia consumption, along with an improvement in the quality of life, as shown by the HIT-6 scores. Regarding the types of headaches, the overall trend was an improvement in any of the headache subgroups. This suggests that one may not need a strict diagnosis of a particular subgroup of headache, such as HARE or TTH, to benefit from the correction of refractive error.

Improvement of headaches and HARE with refractive correction has been reported in several papers. In Gil-Gouveia and Martins’ study, 72.5% of the subjects with headache and refractive error reported improvement in their headaches with adequate correction, headache frequency being significantly reduced regardless of the type of headache [8]. As for surgical correction, complete resolution of headaches after refractive surgery in a migraine patient with anisometropia was reported by Holopainen et al. They suggested that anisometropia may trigger functional changes in visual pathways that are stimulative for migraine [28]. In Lajmi et al.’s study, all headache patients improved after optical correction, prism correction, and orthoptic rehabilitation were indicated. However, these patients were not specifically screened for keratoconus and probably had normal ocular exams.

The relevance of these findings beyond clinical improvement should be highlighted. The reduction in the number of headache-free days carries significant socioeconomic implications, considering the profound socioeconomic losses associated with headache, including medical care, as well as indirect costs due to sickness leave and impaired workplace productivity [2]. A population-based study has shown that individuals with headaches lose the equivalent of 4.2 days of work a year to sickness absence, and that 70% of all work loss takes the form of reduced effectiveness at work [29,30]. It is also possible that the true scale of the impact of migraine and headache is a lot larger, because the majority of these patients do not seek help from their general physician. Moreover, a large proportion of patients (61%) stopped consuming analgesia altogether. This not only carries cost-saving outcomes but results in a decrease in morbidity and reduction in opioid use. Opioid use and comorbid depression or anxiety can also add to morbidity [31].

Several limitations of this study must be acknowledged. First, there is a possibility of selection bias, where patients with headaches may have been more likely to accept participation in our study, and those continuing analgesic abuse may have been more likely to decline participation. Secondly, the small samples sizes of the headache subgroups of TTH and migraine (*n* = 5 each) may affect the sensitivity of the statistical analyses. Furthermore, the prevalence of headache may not be representative of patients with refractive error as a whole, because our headache patient population, although appropriately classified, is from a highly select proportion of the general population—patients with keratoconus in a specialist cornea clinic. We acknowledge the potential placebo effect of surgery by means of TGPRK and CXL towards alleviation of headache. An appropriate next approach will be to compare our data to a gender and age-matched control group.

The findings regarding the impact of headache pre- and post-surgical correction of irregular astigmatism are novel and have not been previously investigated to the best of our knowledge. In our study, patients’ headaches were appropriately classified using the ICHD-3 guidelines. This is contrary to multiple studies investigating the link between refractive error and any headache in the past, where patients of multiple different headache groups were examined. Our results show that patients with keratoconus suffering from headaches are likely to benefit from correction of refractive error and experience a significant improvement in quality of life. Further investigations need to be performed to establish the prevalence of keratoconus in a headache population and the impact of the different options to treat refractive error in these patients, not just a surgical approach. We would anticipate that correction of irregular astigmatism in this population with rigid or scleral lenses would have a similar effect. Therefore, contact lenses should be the first choice of management in patients with keratoconus and HARE, given that this could potentially have the same effect as TGPRK on headache.

In conclusion, our study has demonstrated that improvement of complex refractive error in patients with keratoconus can result in a significant improvement in headache. The improvements in optometric variables following surgical correction were accompanied by an increase in quality of life and, in some cases, complete cessation of analgesic use. Clinicians should be aware of the possible association of keratoconus with headache and the potential therapeutic effect of correcting irregular astigmatism in this group, either with surgery or contact lenses. We would advocate that headache specialists familiarize themselves with keratoconus, its risk factors, and early symptoms to allow detection of this subgroup and referral for specialist treatment.

## Figures and Tables

**Table 1:** Comparison of the preoperative prevalence of refractive errors and anisometropia between groups.

Preoperative Refractive Errors	Overall Headache (*n* = 24)	Migraine (*n* = 5)	HARE (*n* = 14)	TTH (*n* = 5)	Non-Headache (*n* = 16)	*p* Value (Overall Headache vs. Non-Headache)
Myopia						
Mild (−0.5 to −3.0 D)	7 (29.2%)	2 (40%)	3 (21.4%)	2 (40%)	8 (50%)	0.092
Moderate (−3.0 to −6.0 D)	11 (45.8%)	1 (20%)	5 (35.7%)	1 (20%)	3 (18.8%)	0.039
Severe (>6.0 D)	6 (25%)	2 (40%)	4 (28.6%)	2 (40%)	5 (31.3%)	0.667
Astigmatism						
Mild (1.0 to 3.0 D)	10 (41.6%)	2 (40%)	4 (28.5%)	2 (40%)	4 (25%)	0.897
Moderate (3.0 to 6.0D)	7 (29.2%)	2 (40%)	2 (14.3%)	3 (60%)	6 (37.5%)	0.582
Severe (>6.0 D)	7 (29.2%)	1 (20%)	5 (35.7%)	0 (0%)	3 (18.8%)	0.453

HARE: Headache attributed to refractive error, TTH: tension-type headache.

**Table 2:** Postoperative reduction in overall mean number of headache days per week and headache duration.

Group	Headache (Days/Week)						Headache Duration (Minutes)					
	Preoperative		Postoperative		Mean Reduction	*p* Value (Paired *t* Test)	Preoperative		Postoperative		Mean Reduction	*p* Value (Paired *t* Test)
	Mean	Range	Mean	Range			Mean	Range	Mean	Range		
Overall Headache	4.4	1–7	0.5	0–3	3.9	<0.001	108.5	10–360	34.4	0–240	74.1	0.002
(*n* = 24)												
Migraine	3.8	2–7	0.4	0–1	3.4	0.007	125.0	30–240	60.0	0–120	65.0	0.208
(*n* = 5)												
HARE	4.7	1–7	0.6	0–3	4.1	<0.001	115.0	10–360	97.5	5–240	17.5	0.045
(*n* = 14)												
TTH	4.0	1–7	0.2	0–1	3.8	0.030	72.0	15–180	30.0	0–60	42.0	0.072
(*n* = 5)												

*p*-values comparing postoperative and preoperative means for statistical significance (paired *t*-test). HARE: headache attributed to refractive error, TTH: tension-type headache.

**Table 3:** Improvement of HIT-6 scores across all headache groups.

Group	Mean HIT-6 Score		
	Preoperative	Postoperative	*p* Value
Overall headache group (*n* = 24)	62.4	46.6	<0.001
	(40–78)	(36–78)	
Migraine group (*n* = 5)	62.0	45.0	0.006
	(57–66)	(36–60)	
HARE group (*n* = 14)	63.0	47.0	0.001
	(49–78)	(36–78)	
TTH group (*n* = 5)	56.0	42.8	0.176
	(40–70)	(36–58)	

Mean and range are presented. *p*-values comparing preoperative and postoperative values were derived from the paired *t*-test. HIT-6: Headache Impact Test-6, HARE: headache attributed to refractive error, TTH: tension-type headache.

**Table 4:** Improvement of HIT-6 scores across headache subgroups.

HIT-6 Score Categories	Overall Headache Group (*n* = 24)		Migraine Group (*n* = 5)		HARE Group (*n* = 14)		TTH Group (*n* = 5)	
	Preoperative	Postoperative	Preoperative	Postoperative	Preoperative	Postoperative	Preoperative	Postoperative
>60 (disabling pain)	16 (66.7%)	4 (16.7%)	4 (80%)	1 (20%)	10 (71.4%)	3 (21.4%)	2 (40%)	0 (0%)
*p* value	0.001		0.045		0.082		0.157	
56–59 (substantial impact)	2 (8.3%)	1(4.17%)	1 (20%)	0 (0%)	0 (0%)	0 (0%)	1 (20%)	1 (20%)
*p* value	0.006		0.065		n/a		1.000	
50–55	4 (16.7%)	0 (0%)	0 (0%)	0 (0%)	3 (21.4%)	0 (0%)	1 (20%)	0 (0%)
(some impact)								
*p* value	0.001		n/a		0.083		0.065	
<49	2 (8.3%)	19 (79.2%)	0 (0%)	4 (80%)	1 (7.1%)	11 (78.6%)	1 (20%)	4 (80%)
(minimal impact)								
*p* value	<0.001		0.730		0.005		0.045	

*p*-values comparing preoperative and postoperative values were derived from McNemar’s test. HIT-6: Headache Impact Test-6, HARE: headache attributed to refractive error, TTH: tension-type headache, n/a: not available.

**Figure A1:**
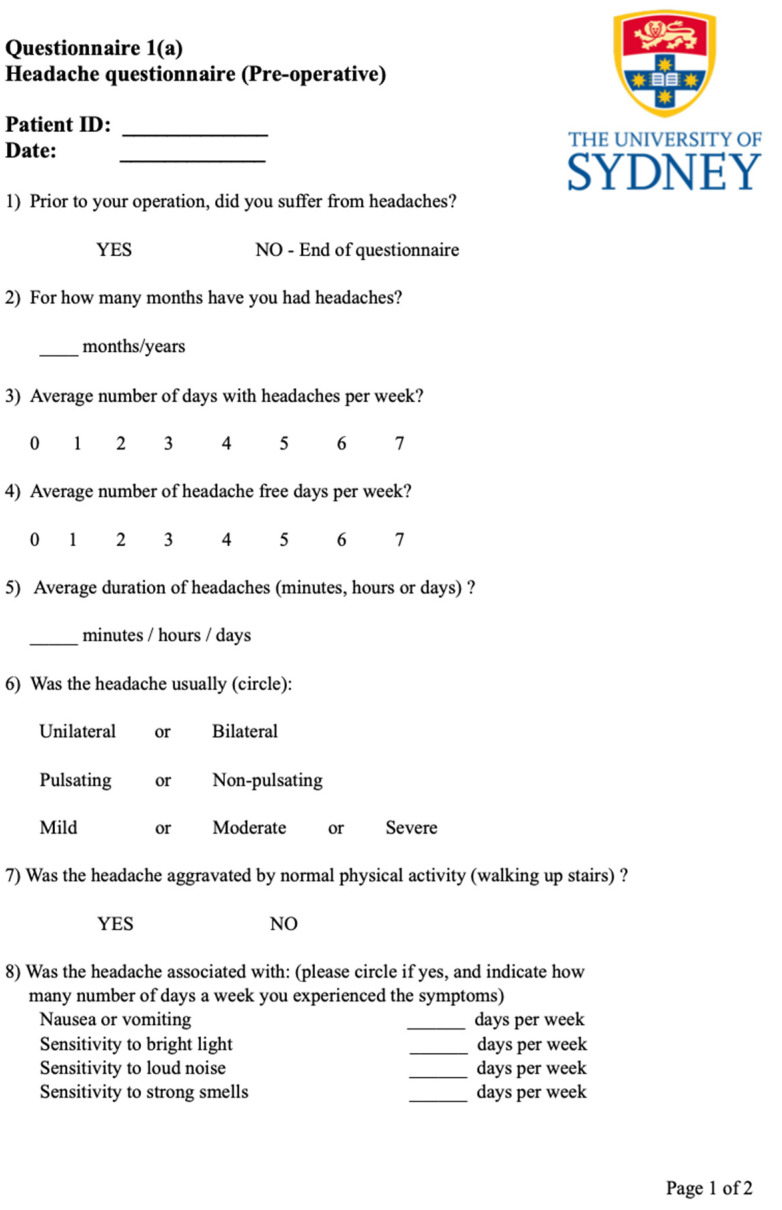

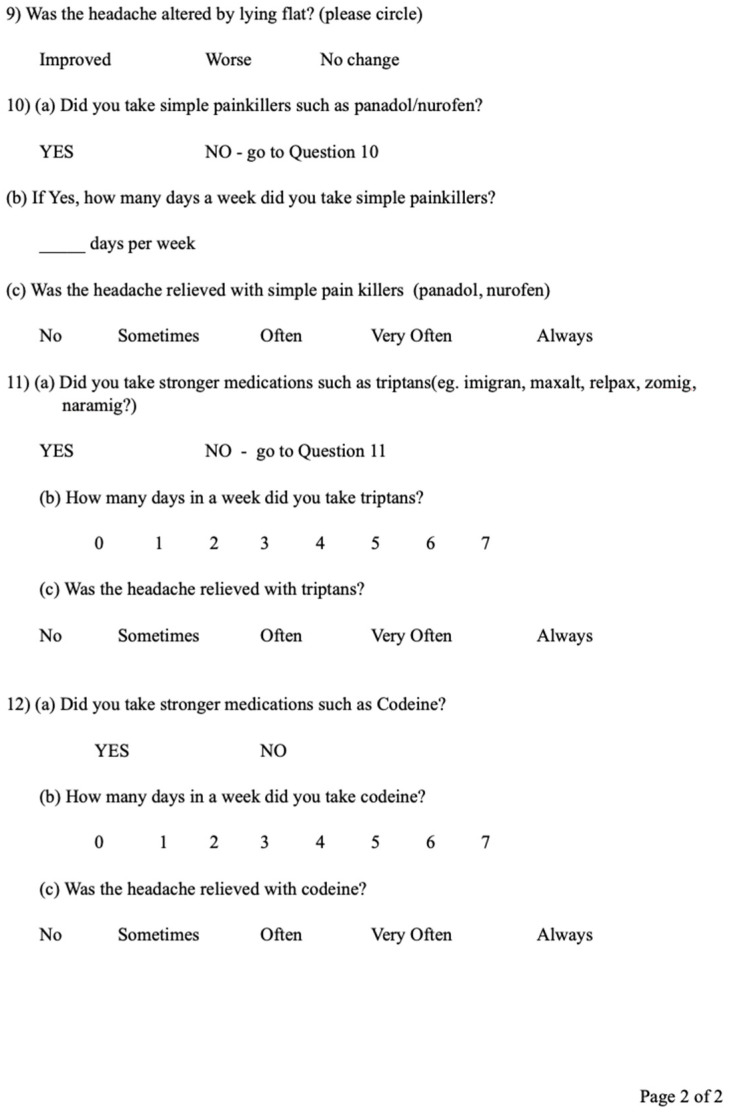
Headache questionnaire.

## Data Availability

The data presented in this study are available on request from the corresponding author.
